# Image quality enhancement in variable-refresh-rate AMOLED displays using a variable initial voltage compensation scheme

**DOI:** 10.1038/s41598-022-09892-5

**Published:** 2022-04-08

**Authors:** Li Jin Kim, Sujin Jung, Hee Jun Kim, Bong Hwan Kim, Kyung Joon Kwon, Yong Min Ha, Hyun Jae Kim

**Affiliations:** 1 School of Electrical and Electronic Engineering, Yonsei University, 50 Yonsei-ro, Seodaemun-gu, Seoul, 03722 Republic of Korea; 2 Mobile Product Development Group, LG Display Co., Ltd, 245 LG-ro, Wollong-myeon, Paju-si, Gyeonggi-do 10845 Republic of Korea

**Keywords:** Electrical and electronic engineering, Engineering

## Abstract

In active matrix organic light emitting diode (AMOLED) displays, when a variable refresh rate is applied and the frame rate changes, the image’s color and luminance quality in AMOLED displays deteriorates. The frequency-dependent cognitive differences were experimentally demonstrated by using 6.76″ AMOLED displays. This phenomenon is dependent on the emission time and the data programming time on the frame rate. This degradation of the image quality during the frequency switch could be prevented by applying a variable initial voltage (V_INI_) to the OLED anode. For a frequency change between 60 and 120 Hz, the measured just noticeable color difference (JNCD) decreased from 7.50 to less than 1.00 in luminance, and from 2.34 to 0.02 in color. As our approach can prevent image quality distortion by utilizing an existing compensation pixel structure without additional compensation steps, it will be a promising technique for improving the picture quality in AMOLED displays.

## Introduction

Due to the evolutionary trend in the cloud era, and high-speed networks that extend beyond 4th to the 5th generation, interconnections between electronic devices are expanding, and the boundaries between devices such as PCs, TVs, and smartphones are disappearing. In addition, we are approaching to an era in which the enjoyment of contents such as games, movies, and sports require high frame rates, as they retrieve data directly from a cloud server in real time without downloading. Thus, the gradually increasing demand for high refresh rate (HRR, ≧ 90 Hz) driving has gone beyond TVs and PCs to smartphones. However, since HRR driving leads to high power consumption and mobile devices are sensitive to the latter, it is therefore necessary to combine both low refresh rates (LRR) and HRR appropriately to compensate for the high HRR power consumption^1^. For example, we can only apply the high-speed driving to moving images such as videos and games, and to scrolling images. To reduce the increasing power consumption raised from HRR driving, a still image is driven by a conventional normal refresh rate (NRR) of 60 Hz, and LRR also could be applied in standby modes such as an always on display (AoD). However, a change in frame rate can deteriorate image quality of active matrix organic light emitting diode (AMOLED) displays, as the threshold voltage compensation^1–4^ and the OLED charging depend on the programming time^5–8^. In this study, we characterized the image degradation by measuring color and luminance deviations at various frequencies. Furthermore, a new driving method is proposed and it can solve the problem without additional external or internal compensation steps, or modification of the pixel structure.

## Experimental section

For the measurements, 6.76″ sized 1,344 × 2,772 resolution AMOLED displays with 7T1C compensation pixel structures of low temperature polysilicon (LTPS) thin-film transistors (TFTs) backplane were used. The size of driving TFTs is width/length = 3.0/16.3 μm. KONICA MINORTA's CA-410 was used for optical measurement. The CA-410 was subjected to regular calibration and gage repeatability and reproducibility (R&R) through a standard light source. The measurements were performed at the center of the sample after aging AMOLED displays for 10 min at the maximum luminance to stabilize them. Considering the hysteresis characteristics of TFTs^9,10^, luminance and color were measured after fixing luminance at each frequency condition, and then this process was repeated for all other luminance conditions. 10 samples were measured in consideration of reproducibility and repeatability, and the average value of the measured results was shown as the experimental result. The minimum and maximum luminance of the luminance conditions were 2 and 600 nit respectively. The color space coordinates (u′, v′) in the system are defined by the commission internationale de l'Éclairage (CIE) 1976^11^; the target of color u′ and v′ are 0.193 and 0.459 respectively. To numerically analyze luminance (L) and color (u′, v′) fluctuations, the formulae ΔL = (L_2_ − L_1_/L_2_) × 100 and Δu′v′ = ((u′_1_ − u′_2_)^2^) + ((v′_1_ − v′_2_)^2^))^0.5^ were used. The just noticeable color difference (JNCD)-a measure for evaluating the color accuracy of a display^12,13^-was used for comparison. A JNCD value of less than 1 is currently considered excellent. A JNCD value of 1 corresponds to a 0.004 in color (Δu′v′)^14,15^. In addition, the result of deriving the luminance deviation corresponding to 0.004 color deviation through the experimental method is at the level of 2%. Furthermore, the JNCD value increases proportionally with the amount of deviation. In this experiment, we defined 60 Hz as NRR and 90 Hz or 120 Hz as HRR. The corresponding 1-frame and 1-horizontal time for each frequency are 16.7 ms/4.5 μs, 11.1 ms/3.0 μs, and 8.3 ms/2.1 μs respectively. The 4.6 V ELVDD and the − 3.0 V ELVSS were applied to drive the OLED respectively. The typical V_INI_ was − 2.8 V. V_GH_ and V_GL_ were applied with 7.5 V and − 8.5 V to drive the gate in panel (GIP) driving, respectively. In addition, our measurement results were cross-confirmed with the simulation using Silvaco's Smart Spice Tool.

## Discussion

**Figure 1:**
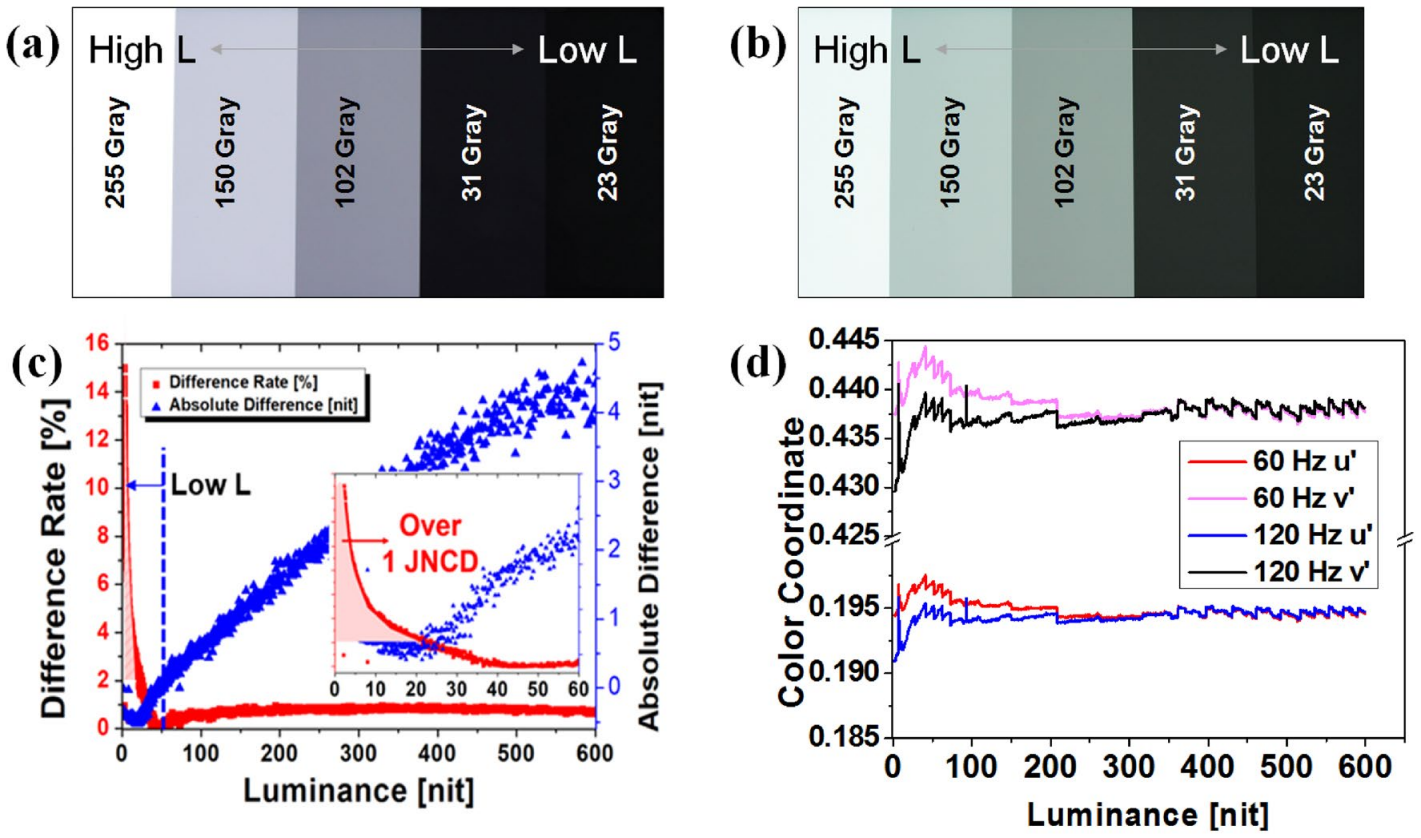
T the gamma scale for frequencies of (**a**) 60 Hz, and (**b**) 120 Hz, without compensation. The differences in (**c**) luminance and (**d**) color, between frequencies of 60 Hz and 120 Hz.

Figure 1a and b show the gray scale bars displayed by the AMOLED at frequencies of 60 and 120 Hz respectively, with the gray 255 luminance corresponding to 50 nit. Comparing Fig. 1a and b, we confirmed that the color and luminance changed significantly between two corresponding frequency and gray conditions. To analyze the image deterioration numerically, we measured the differences in luminance and colors between 60 and 120 Hz frequency conditions. In Fig. 1c, the right axis shows the absolute difference in luminance, and the left axis shows the difference rate expressed in percentage. Even though the absolute difference is large for high luminance, the difference rate increases as the luminance decreases. As human vision concerning luminance has non-linear characteristics, the cognition of luminance is more sensitive to the difference rate of luminance rather than to the absolute difference^16^. Hence, we mainly used the difference rate to describe the results.

In Fig. 1c, it is confirmed that the value of JNCD exceeds 1 under luminance of 20 nit, and increased further as the luminance decreases. We shall explain the root causes of the difference between the two frequency conditions separately in the high and low luminance regions. In the high luminance region, the main cause of luminance deterioration is data leakage, which inevitably occurs in the storage capacitor holding data during 1 frame time. After data programming for the emission, the power of the capacitor is floated, and data leakage occurs over the entire emission period until new data are programmed. Since more leakage occurs during the longer frame time of LRR. Compared to HRR, LRR luminance is lower than HRR luminance in the high-luminance region.

On the other hand, the low-luminance region is driven by a small current, so the OLED saturation characteristics are mainly affected by the parasitic capacitance of the OLED. With HRRs, current flow during OLED charging is disturbed heavily by the parasitic capacitor due to the relatively short emission time compared to LRR. Accordingly, in the low-luminance region, HRR luminance cannot reach the target luminance, thus, HRR luminance is lower than the NRR.

As shown in Fig. 1c, luminance of less than 50 nits has a negative absolute difference value. This phenomenon is also evident in the color variations in Fig. 1d. In general, the emission efficiencies of red and green OLEDs are higher than that of blue OLEDs. Therefore, in the low-luminance region, the color fluctuates in the direction of increasing values of both u' and v' under HRR, resulting in the color variation shown in Fig. 1b.

**Figure 2:**
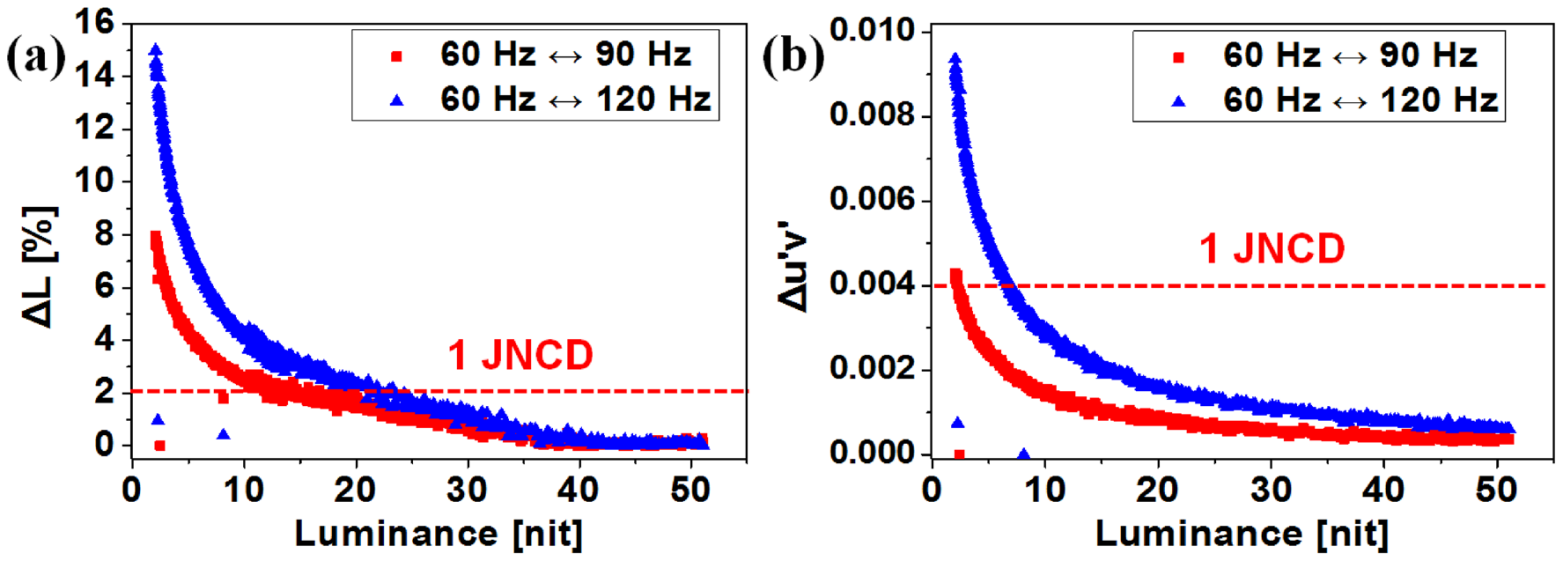
Differences in (**a**) luminance and (**b**) color under a frequency change from 60 to 90 Hz and 60 Hz to 120 Hz without V_INI_ compensation.

Figure 2a and b show the difference in luminance and color after the frequency changes from 60 to 90 Hz, and from 60 to 120 Hz, respectively. We focused on the low luminance region (< 50 nit) where huge differences can be noticed in Fig. 1. The differences in luminance and color between two frequencies increase as a result of the shorter emission and data programming time.

**Figure 3:**
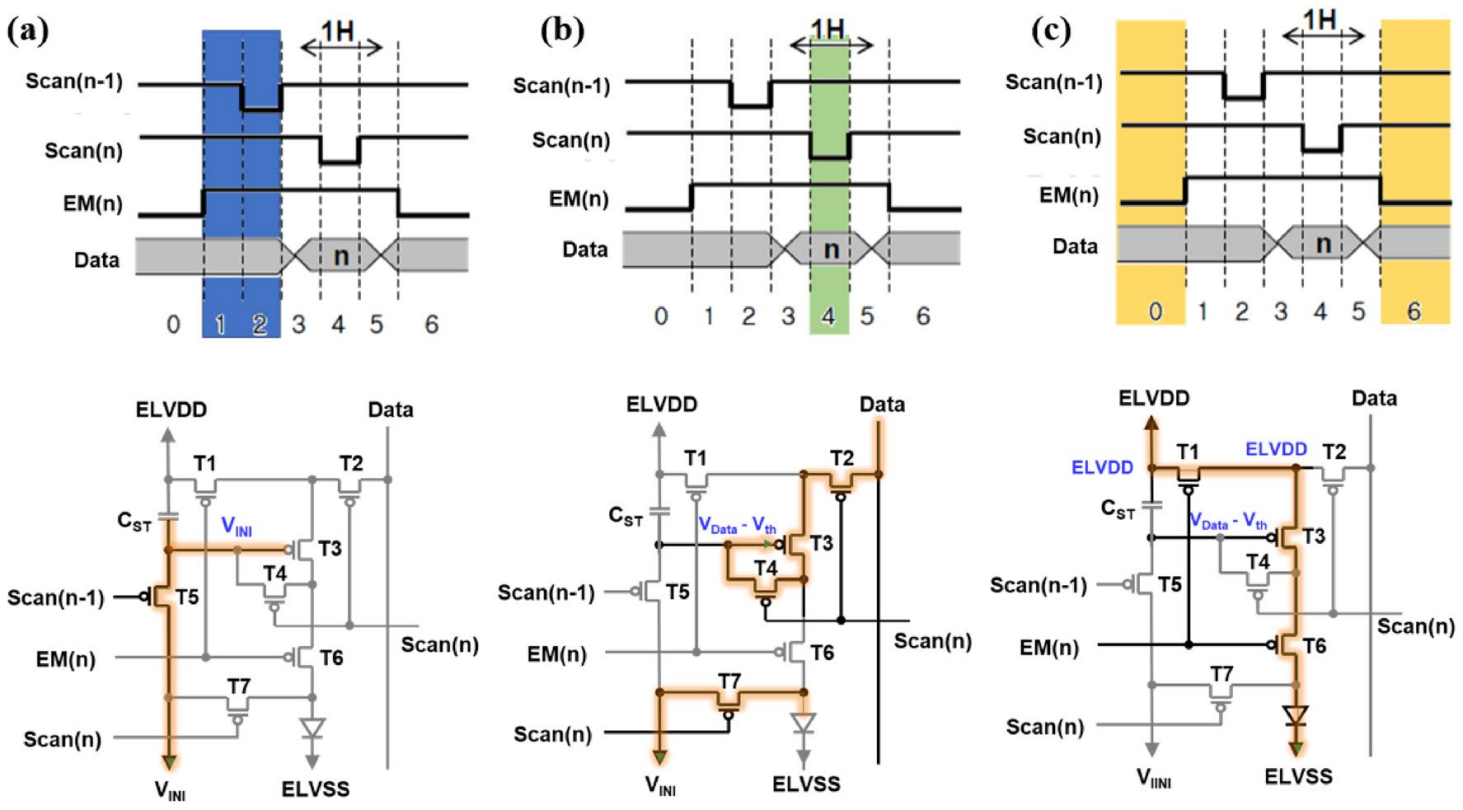
Driving sequence of 7T1C compensation pixels: (**a**) initialization step, (**b**) sampling step, (**c**) emission step.

Before explaining the detail mechanism of this phenomenon, the operation sequence of 7T1C compensation structure is described in Fig. 3. First step is (a) initialization, where T_1_ and T_6_ are turned off by EM signal. T_5_ is turned on by Scan[n − 1], which means the gate node of driving TFT and storage capacitor are initialized. Next step is (b) sampling. T_2_, T_3_, T_4_, and T_7_ are turned on by the Scan[n] signal. Therefore, the threshold voltage (V_th_) of the driving TFT is stored in the storage capacitor and the OLED anode is initialized. The final step of pixel driving is (c) emission. T_1_, T_3_, and T_6_ are turned on by the EM signal, so compensated data voltage is stored in storage capacitor and maintains the emission state. And the formula for the current flowing through the OLED and V_th_ compensation are shown in Eq. (1), where k is the parameter determined by the mobility µ, the oxide capacitance C_ox_, and channel width (W) and length (L). Therefore the I_OLED_ becomes independent of the V_th_ of driving TFT (T3).

1 $$ \begin{aligned} I_{OLED} = k\left( {V_{GS} - V_{th} } \right)^{2} & = k\left( {ELVDD - V_{Data} + V_{th} - V_{th} } \right)^{2} \\ & = k\left( {ELVDD - V_{Data} } \right)^{2} \\ \end{aligned} $$

**Figure 4:**
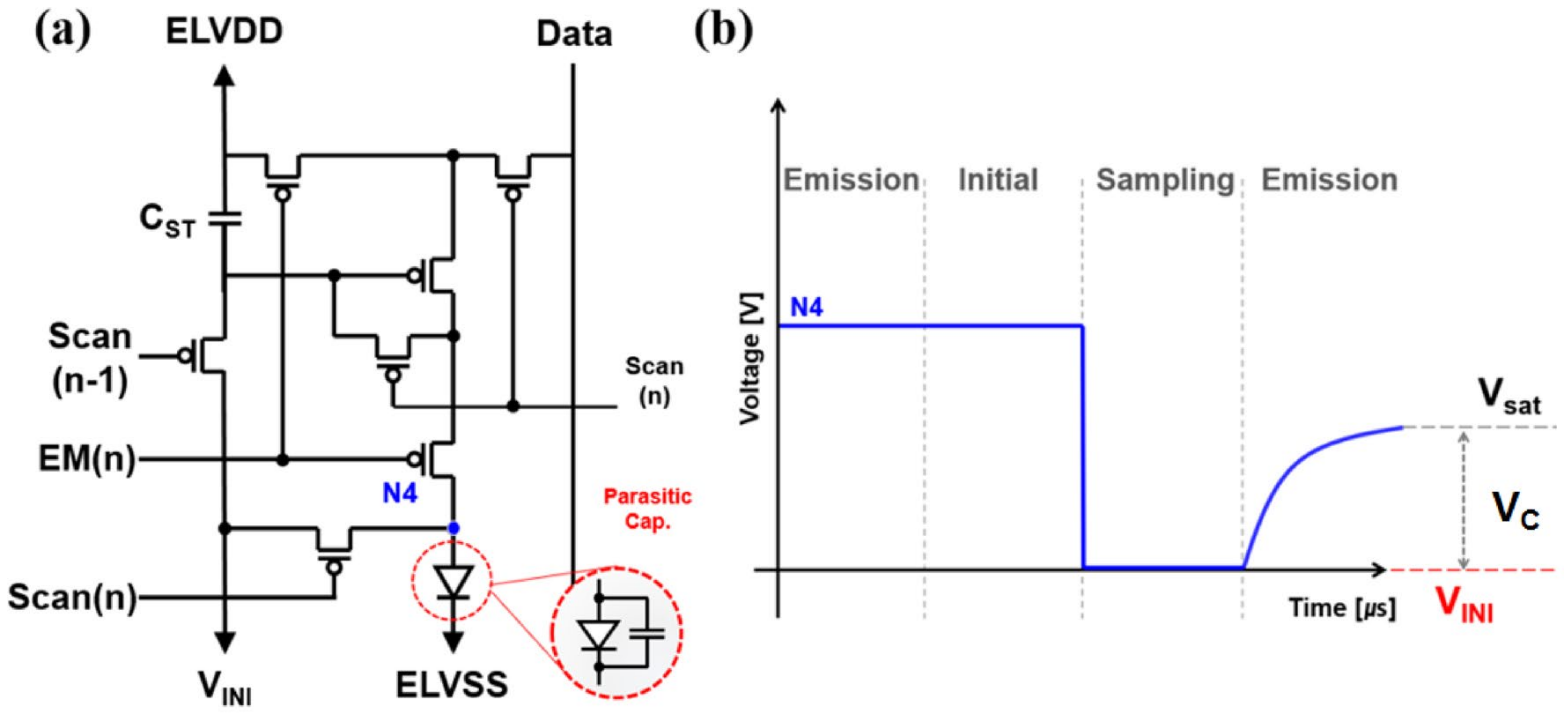
(**a**) Schematic of a 7T1C pixel circuit. (**b**) A schematic diagram for the OLED anode node voltage described by the compensation process.

Figure 4 shows the mechanism that causes image distortion to occur more often in the low luminance region as the frequency is increased, and how this can be prevented. Figure 4a shows a p-type LTPS 7T1C compensation pixel circuit diagram. During the initialization process, the driving TFT is controlled by the V_INI_. The V_INI_ resets the gate node and the storage capacitor while simultaneously resetting the anode (N_4_) voltage^3^. The V_INI_ resets the gate node and the storage capacitor and simultaneously resets the anode (N_4_) voltage. After the programming step, the N4 node has the V_INI_ in a floating state. After the programming step, the N4 node is maintained at the V_INI_ until the emission, and then emitting as the current is applied to the OLED in the emission period. When the OLED is in saturation state, to emit light, it is necessary to apply a voltage to charge the OLED, which can reach the saturation state from the initial state. Since V_INI_ is generally negative, the relationship among the voltage for charging the OLED (V_C_), the initial voltage, and the saturation voltage (V_SAT_) can be expressed as the following:

2 $$ V_{C} = V_{SAT} - V_{INI} $$

The V_SAT_ can be expressed as below formula, which contains the current of specific luminance (I_L_), emission time (T_E_) and OLED capacitance (C_OLED_). Then the voltage V_C_ required for emission can be replaced as the following:

3 $$ V_{C} = \frac{{I_{L} { } \times { }T_{E} }}{{C_{OLED} }} - V_{INI} $$

Due to insufficient current in the low luminance region, charging is not efficient. HRR, given its short sampling and emission time, its charging capacity is weaker than LRR’s. It is possible to say that the fluctuation in the anode voltage originates from the starting value of V_INI_ for the anode voltage, as shown in Fig. 4b. At each frequency, to match the charging characteristics to the target V_SAT_, V_C_ should be increased by a variable V_INI_ value.

**Figure 5:**
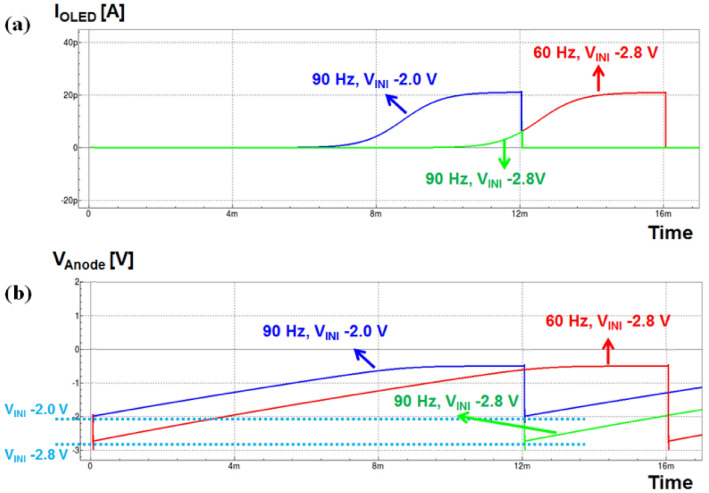
Simulation results for (**a**) OLED pixel current and (**b**) anode voltage in various V_INI_ at 60 and 90 Hz conditions.

Figure 5 shows the validation for mechanism and improvement through simulation. From the simulation results, it can be seen that when the same V_INI_ was applied under 90 Hz HRR and 60 Hz NRR conditions. The OLED current is significantly reduced in HRR condition due to the charging delay. On the other hand, when the V_INI_ is raised from − 2.8 to − 2.0 V under the 90 Hz condition, it is confirmed that in 1 frame the current amounts under 90 Hz is similar to 60 Hz. Through the simulation results, it was confirmed that the OLED current deviation during frequency changes could be compensated through V_INI_ control securing the anode voltage charge.

**Figure 6:**
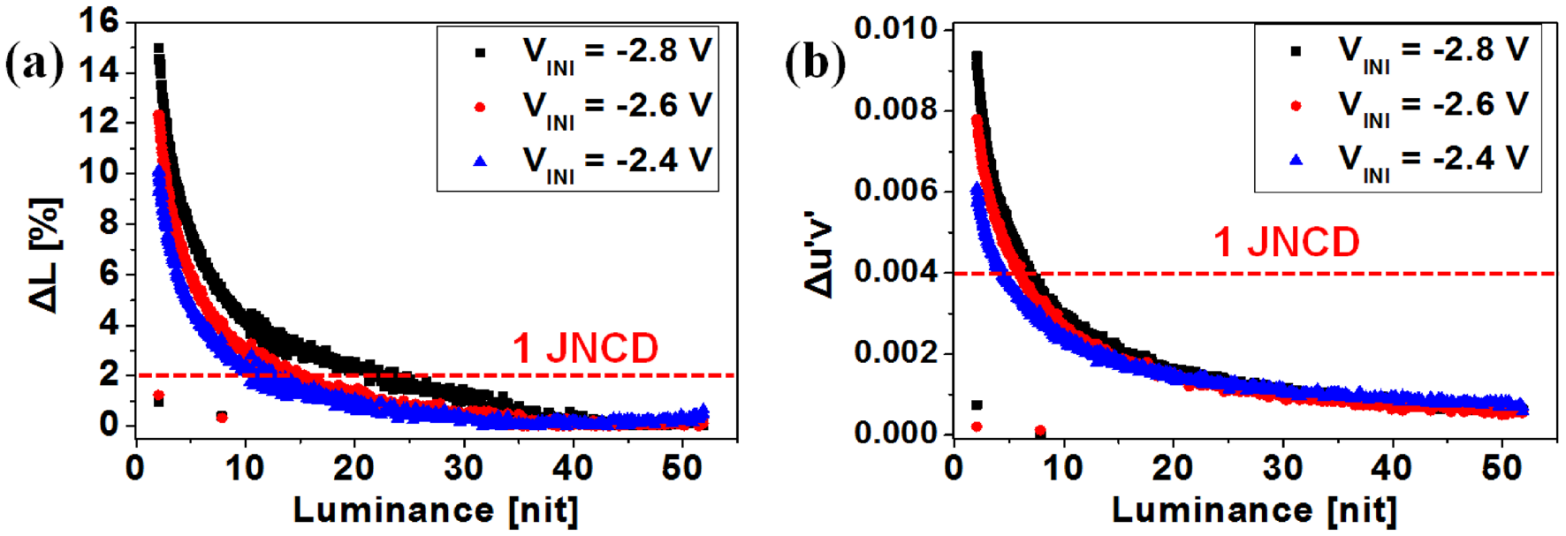
(**a**) The luminance and (**b**) color differences for a frequency change from 60 to 120 Hz, with fixed initial voltages of − 2.8, − 2.6, and − 2.4 V.

Figure 6 shows the difference rate between 60 and 120 Hz frequency, in different V_INI_ conditions. As the V_INI_ increases, the fractional differences in the quantities become smaller. As can be seen in Fig. 6a and b, depending on the V_INI_ setting, luminance and color difference were improved from 7.5 (15%)/2.25 (0.009) to 5 (10%)/1.5 (0.006) respectively. Image quality distortion will still occur, however, due to the different influence of the parasitic capacitance at each frequency. For further improvement, it will be necessary to adjust the saturation characteristics by applying the V_INI_ individually for each frequency, as mentioned before.

**Figure 7:**
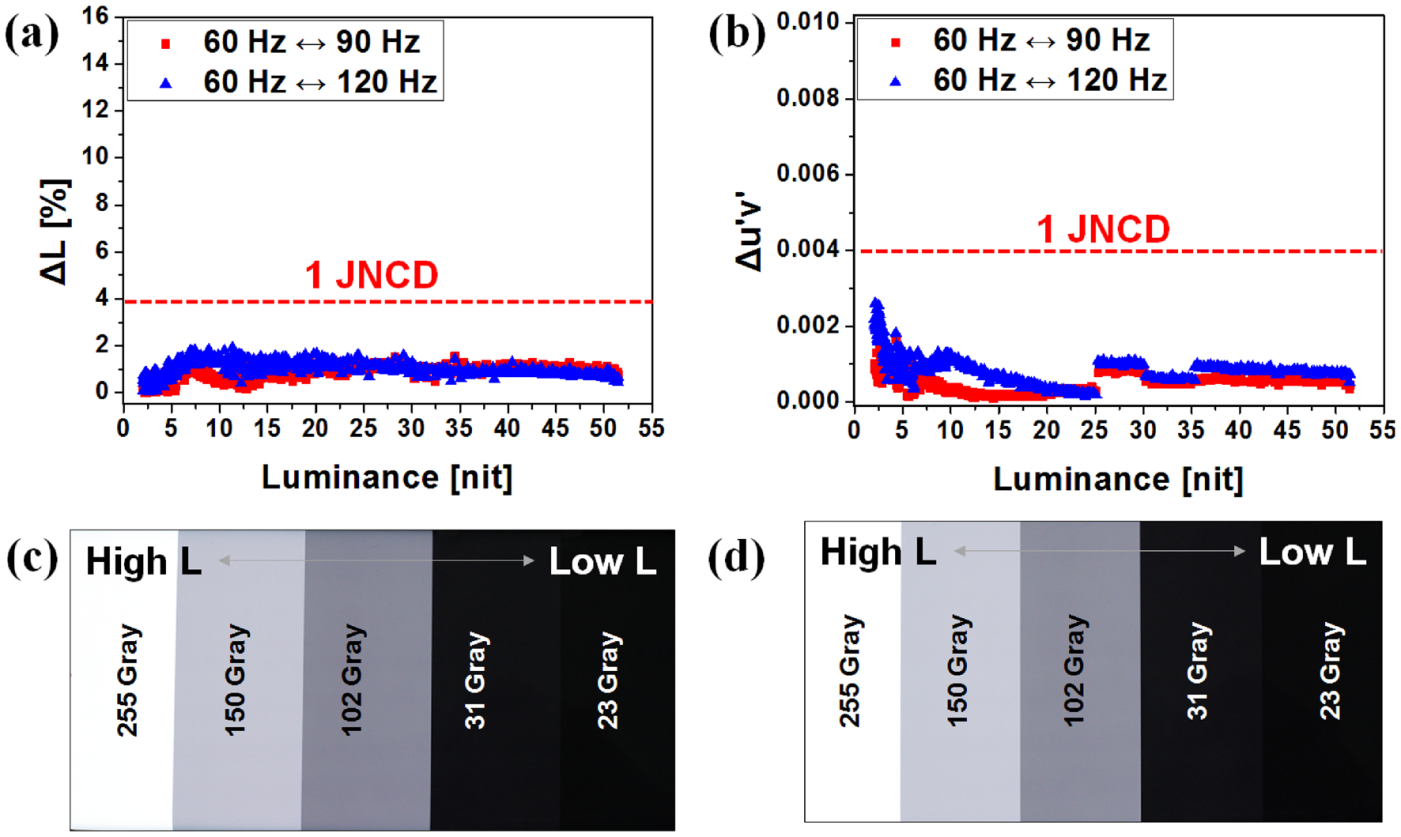
(**a**) The luminance and (**b**) color differences under frequency changes. The initial voltages were: − 2.8 V for 60 Hz, − 2.6 V for 90 Hz, and − 2.4 V for 120 Hz. The displayed gamma scales for (**c**) 60 Hz and (**d**) 120 Hz, after V_INI_ compensation was applied.

**Figure 8:**
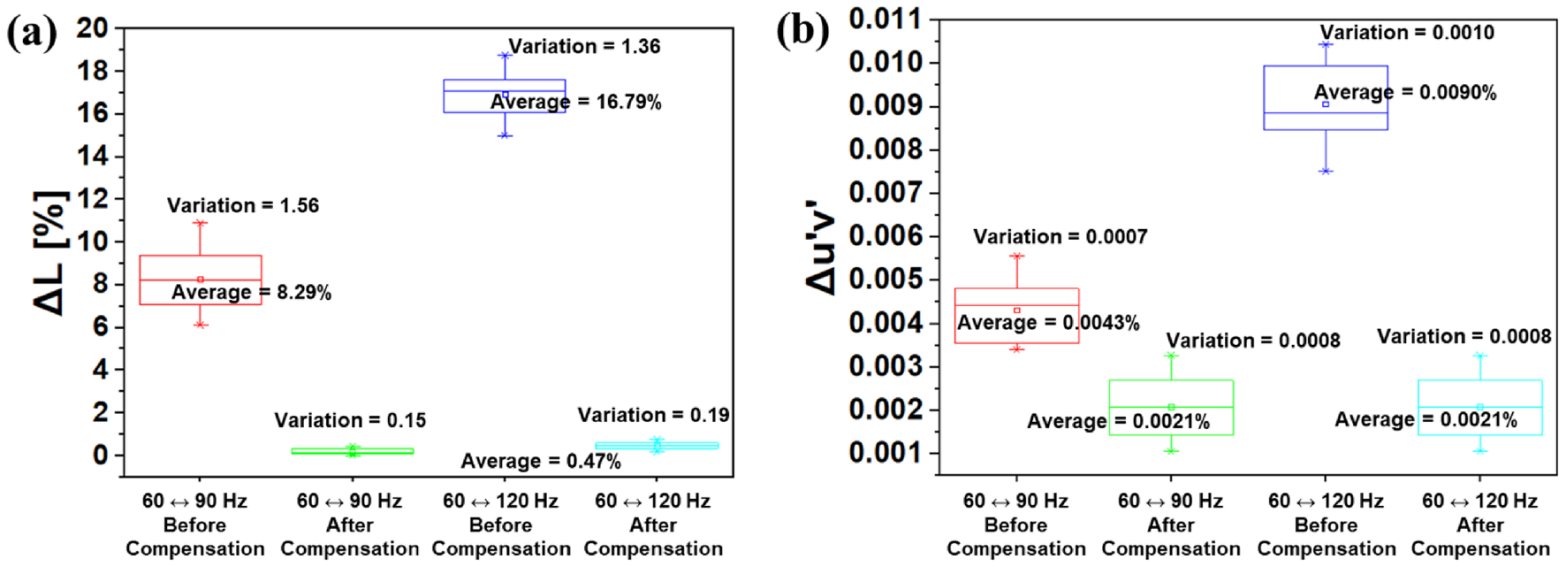
Box plot data for (**a**) luminance and (**b**) color differences showing their variation before and after the initial voltage compensation evaluated with 10 samples each.

While in Fig. 6, a fixed V_INI_ has been applied for a frequency change between 60 and 120 Hz, we investigated the results when different V_INI_ were used for different frequencies: − 2.8 V for 60 Hz, − 2.6 V for 90 Hz, and − 2.4 V for 120 Hz. The results are shown in Fig. 7a and b. After applying voltage compensations, both color and luminance fluctuations decrease to less than 1 JNCD. In Fig. 7c and d, various gray scales displayed on the 6.76″ AMOLED display under different frequency conditions of 60 and 120 Hz, respectively, are shown. After the variable V_INI_ is applied, no corruption in the image quality has occurred even in the case of 120 Hz as well as the case of 60 Hz. Figure 8 shows the box plot data for luminance and color difference before and after the V_INI_ compensation, using 10 samples for each case, to confirm the reproducibility of the proposed method. Reproducibility was verified as can be indicated from the figure where the variations were significantly reduced once the compensation method was applied.

## Conclusion

The causes for the degradation in image quality of AMOLED display in variable refresh rate have been examined. At high driving frequencies, we confirm that the effect of parasitic capacitance is significant in the low luminance region. This degradation of image quality in low luminance, which is particularly noticeable to human eyes, can be prevented by applying individual V_INI_ values to the OLED without additional compensation steps. We demonstrated the effectiveness of this technique by applying it to a 6.76″ sized AMOLED display panel. When switching frequency from 60 to 120 Hz, the luminance distortion decreased from 7.5 to less than 1 JNCD, and the color quality was improved from 2.34 to 0.02 JNCD, which could not be achieved with the traditional compensation method.
